# Erianin, a novel dibenzyl compound in Dendrobium extract, inhibits lung cancer cell growth and migration via calcium/calmodulin-dependent ferroptosis

**DOI:** 10.1038/s41392-020-0149-3

**Published:** 2020-05-08

**Authors:** Peng Chen, Qibiao Wu, Jiao Feng, Lili Yan, Yitian Sun, Shuiping Liu, Yu Xiang, Mingming Zhang, Ting Pan, Xiaying Chen, Ting Duan, Lijuan Zhai, Bingtao Zhai, Wengang Wang, Ruonan Zhang, Bi Chen, Xuemeng Han, Yicong Li, Liuxi Chen, Ying Liu, Xingxing Huang, Ting Jin, Wenzheng Zhang, Hong Luo, Xiaohui Chen, Yongqiang Li, Qiujie Li, Guohua Li, Qin Zhang, Lvjia Zhuo, Zuyi Yang, Huifen Tang, Tian Xie, Xiaoping Ouyang, Xinbing Sui

**Affiliations:** 1 Holistic Integrative Pharmacy Institutes and Department of Medical Oncology, the Affiliated Hospital of Hangzhou Normal University, College of Medicine, Hangzhou Normal University, Hangzhou, Zhejiang China; 2 Institute of Chinese Materia Medica, Shanghai University of Traditional Chinese Medicine, Shanghai, 201203 China; 3 Key Laboratory of Elemene Class Anti-cancer Chinese Medicine of Zhejiang Province, Hangzhou Normal University, Hangzhou, Zhejiang, China; 4 State Key Laboratory of Quality Research in Chinese Medicines, Faculty of Chinese Medicine, Macau University of Science and Technology, Macau, P.R. China; 5 Zhejiang Chinese Medicinal University, Hangzhou, China; 6 Dongzhimen Hospital, Beijing University of Chinese Medicine, Beijing, China; 7 Department of Medical Oncology, Zhejiang University, Hangzhou, China; 8 Department of Hematology and Oncology, the Affiliated Hospital of Hangzhou Normal University, College of Medicine, Hangzhou Normal University, Hangzhou, Zhejiang, China; 9 Radiation Detection Research Center, Northwest Institute of Nuclear Technology, Xi’an, China; 10 Jiangsu Key Laboratory for Pharmacology and Safety Evaluation of Chinese Materia Medica, School of Pharmacy, Nanjing University of Chinese Medicine, Nanjing, China

**Keywords:** Oncology, Lung cancer

## Abstract

Ferroptosis, a novel form of programmed cell death, is characterized by iron-dependent lipid peroxidation and has been shown to be involved in multiple diseases, including cancer. Stimulating ferroptosis in cancer cells may be a potential strategy for cancer therapy. Therefore, ferroptosis-inducing drugs are attracting more attention for cancer treatment. Here, we showed that erianin, a natural product isolated from *Dendrobium chrysotoxum Lindl*, exerted its anticancer activity by inducing cell death and inhibiting cell migration in lung cancer cells. Subsequently, we demonstrated for the first time that erianin induced ferroptotic cell death in lung cancer cells, which was accompanied by ROS accumulation, lipid peroxidation, and GSH depletion. The ferroptosis inhibitors Fer-1 and Lip-1 but not Z-VAD-FMK, CQ, or necrostatin-1 rescued erianin-induced cell death, indicating that ferroptosis contributed to erianin-induced cell death. Furthermore, we demonstrated that Ca^2+^/CaM signaling was a critical mediator of erianin-induced ferroptosis and that blockade of this signaling significantly rescued cell death induced by erianin treatment by suppressing ferroptosis. Taken together, our data suggest that the natural product erianin exerts its anticancer effects by inducing Ca^2+^/CaM-dependent ferroptosis and inhibiting cell migration, and erianin will hopefully serve as a prospective compound for lung cancer treatment.

## Introduction

Lung cancer is the most common cause of cancer death around the world.^1^ Surgery is still the key approach used for lung cancer therapy; however, a large number of patients die due to recurrence and drug treatment failure.^2^ Despite recent advances in the development of new chemotherapeutic agents, molecularly targeted drugs, and antibodies that block immune checkpoints, many obstacles remain.^3,4^ Therefore, identifying new anticancer strategies besides chemotherapy, radiation, and immunotherapy will be critical if we hope to improve cure rates for patients with advanced lung cancer.

Erianin, a natural product isolated from *Dendrobium chrysotoxum Lindl*, has been reported to exert antitumor effects on several cancer types. Wang et al.^5^ demonstrated that erianin could be a promising drug for the treatment of osteosarcoma, as it induced G2/M cell cycle arrest and triggered cell death via the ROS/JNK signaling pathway. Erianin is also considered a promising natural agent for the treatment of human nasopharyngeal carcinoma that acts by inducing cell apoptosis through the ERK pathway.^6^ Moreover, erianin was shown to exhibit antitumor activity in bladder cancer cells via the JNK pathway.^7^ However, the role of erianin in lung cancer remains unclear.

Ferroptosis is a novel form of cell death that is characterized by high iron levels and the accumulation of lipid-reactive oxygen species (ROS) within the cell.^8^ It is known that ferroptosis is genetically, biochemically, and morphologically distinct from other already established forms of cell death, including necrosis, apoptosis, and autophagy.^9,10^ Recently, it has been demonstrated that ferroptosis is also a critical regulator of tumor growth.^8,11^ However, the role of ferroptosis in lung cancer has remained unexplored.

In this research, we investigated the effect of erianin on the viability of two different lung cancer cell lines and found that erianin induced cell death and G2/M-phase arrest and inhibited the migration of lung cancer cells. Next, we demonstrated for the first time that ferroptosis contributed to erianin-induced cell death both in vitro and in vivo, which was accompanied by ROS accumulation, GSH depletion, and lipid peroxidation. Subsequently, we showed that Ca^2+^/CaM signaling was a critical mediator of erianin-induced ferroptosis. Taken together, our results suggest that the natural product erianin exerts its anticancer effects by inducing calcium/calmodulin-dependent ferroptosis and inhibiting metastasis in lung cancer cells.

## Results

### Erianin triggers cell death, inhibits cell proliferation, and promotes cell cycle arrest in G2/M in lung cancer cells

**Fig. 1:**
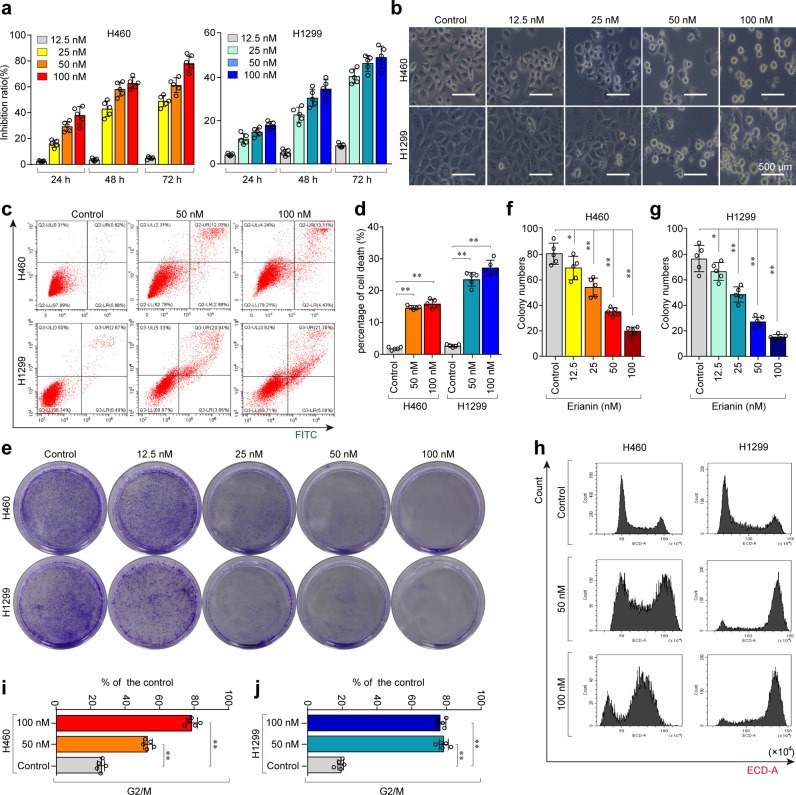
Erianin inhibits cell proliferation and triggers cell death and G2/M cell cycle arrest in lung cancer cells. **a** Cell viability was measured using the CCK-8 assay. **b** Representative cell morphological changes are shown. **c**, **d** Representative results of annexin V/FITC/PI staining and quantitative analysis, **p < 0.01. **e** Monolayer culture; quantitative analyses of colony numbers are shown (**f**, **g**), *p < 0.05, **p < 0.01. **h** Representative results of cell cycle and quantitative analyses (**i**, **j**), *p < 0.05, **p < 0.01

To observe the cytotoxicity and inhibitory effects of erianin in lung cancer cells, the H460 and H1299 cell lines were treated with different concentrations of erianin for different times. Cell counting kit-8 (CCK-8) assays showed that erianin increased the inhibition of the growth of lung cancer cells (Fig. 1a, b). To determine the effect of erianin on apoptosis and cell death, an Annexin V–FITC dual staining assay was performed by flow cytometry. As a result, a significantly increased number of dead cells was observed in these lung cancer cells after treatment with erianin (Fig. 1c, d).

To determine the anti-proliferation effect of erianin, a colony-formation assay was performed. The results showed that erianin treatment significantly suppressed the colony-formation efficiency (Fig. 1e–g), indicating that erianin treatment inhibited the proliferation of lung cancer cells.

To further confirm whether erianin inhibited cell proliferation by inducing cell cycle arrest, flow cytometry was performed. The results showed that erianin promoted G2/M-phase arrest, which was accompanied by a decrease in the number of cells in the G0/G1 and S phases (Fig. 1 h–j). Taken together, the results showed that erianin treatment potently reduced cell viability and induced cell death and G2/M-phase arrest in lung cancer cells.

### Erianin suppresses the migration of lung cancer cells

**Fig. 2:**
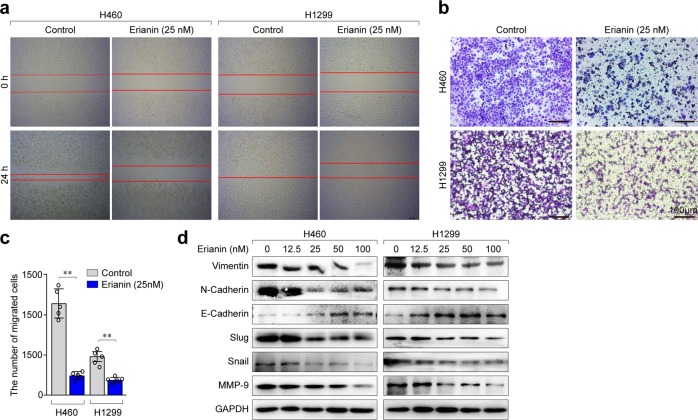
Erianin suppresses the migration of lung cancer cells. **a** Representative results of wound-healing assays. **b** Transwell migration assay with the 24-well Transwell system and quantitative analysis (original magnification: ×100). **c** The numbers of migrated cells were counted in five representative high-power fields per Transwell plate, **p < 0.01. **d** The expression of several key cell metastatic signal regulators, vimentin, E-cadherin, N-cadherin, slug, snail, and MMP-9, were examined by western blotting after treatment with erianin for 24 h

To investigate whether erianin affects cell migration, wound-healing assays were performed. The results showed that treatment with 25 nM erianin significantly reduced migration compared with that in controls (Fig. 2a). Next, a Transwell assay was performed to further confirm the negative effect of erianin on invasive cell migration. As shown in Fig. 2b, c, erianin significantly suppressed lung cancer cell migration after exposure for 24 h. We next detected the expression of several metastatic phenotype markers in lung cancer cells. We found that erianin suppressed the migration of lung cancer cells by downregulating the mesenchymal markers vimentin, N-cadherin, slug, snail, and MMP-9 and upregulating the epithelial marker E-cadherin (Fig. 2d). These data suggest that erianin might suppress lung cancer cell migration by inhibiting epithelial–mesenchymal transition (EMT).

To determine the relationship between erianin-induced ferroptosis and metastasis, the ferroptosis inhibitor deferoxamine (DFO) was used to treat the lung cancer cells. As a result, the inhibition of erianin-induced ferroptosis promoted cell migration, indicating that ferroptosis could suppress the migration of lung cancer cells (Supplementary Fig. S1).

### Ferroptosis contributes to erianin-induced cell death in lung cancer cells

**Fig. 3:**
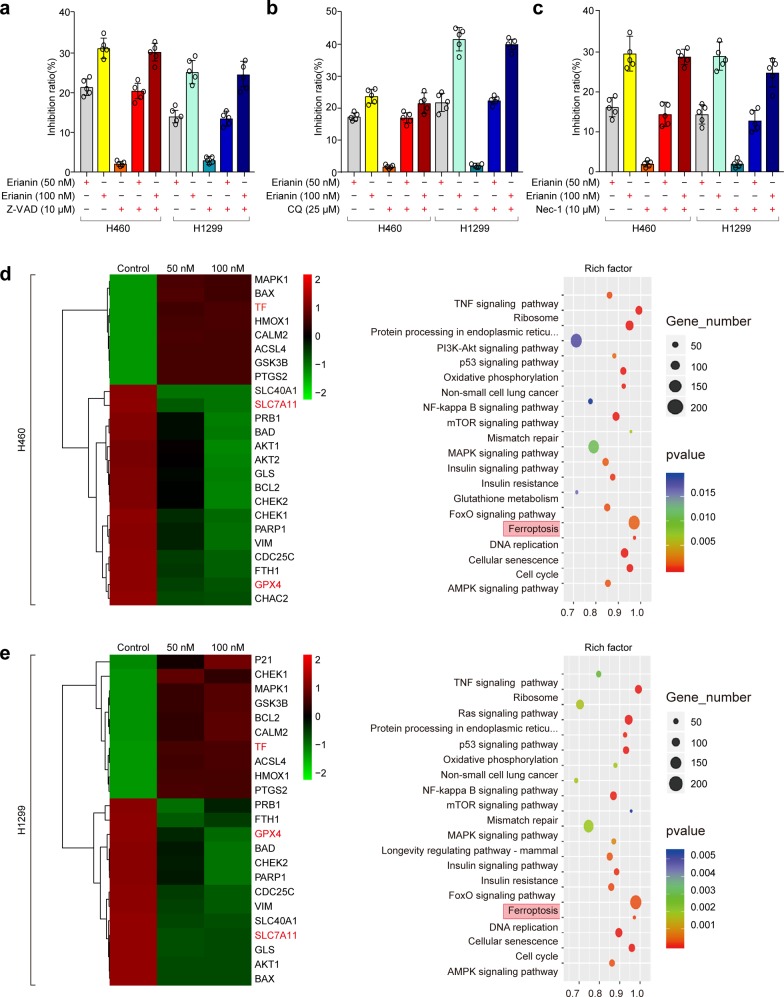
The effect of erianin alone or in combination with other cell death inhibitors on the inhibition of growth of lung cancer cells. **a** H460 and H1299 cells were treated with erianin with or without Z-VAD-FMK for 24 h, and the inhibition of growth was assayed. **b** H460 and H1299 cells were treated with erianin with or without CQ for 24 h, and the inhibition of growth was assayed. **c** H460 and H1299 cells were treated with erianin with or without necrostatin-1 for 24 h, and the inhibition of growth was assayed. **d** Heatmap showing the mRNA and KEGG pathway enrichment analyses in H460 cells. **e** Heatmap showing the mRNA and KEGG pathway enrichment analyses in H1299 cells

As shown in Fig. 1c and Supplementary Fig. S2, annexin-V/propidium iodide (AV/PI) staining and western blotting suggested the occurrence of non-apoptotic cell death after erianin treatment. To determine the role of erianin-induced cell death, several cell death inhibitors were utilized. Treatment with Z-VAD-FMK (a pancaspase inhibitor), chloroquine (CQ, a potent inhibitor of autophagy), or necrostatin-1 (a potent inhibitor of necroptosis) did not protect against erianin-induced cell death in these cells (Fig. 3a–c), indicating that other forms of cell death may have occurred.

Next, the differentially expressed genes in erianin-treated and untreated lung cancer cells were determined by transcriptome analysis. Heatmap analysis revealed that glutathione peroxidase 4 (GPX4, a negative target of ferroptosis) was consistently downregulated in H460 (Fig. 3d) and H1299 (Fig. 3e) cells. Furthermore, we performed KEGG pathway enrichment analyses and found that the ferroptosis pathway was enriched (Fig. 3d, e). Therefore, we postulated that ferroptosis might be a key determinant of erianin-induced cell death.

**Fig. 4:**
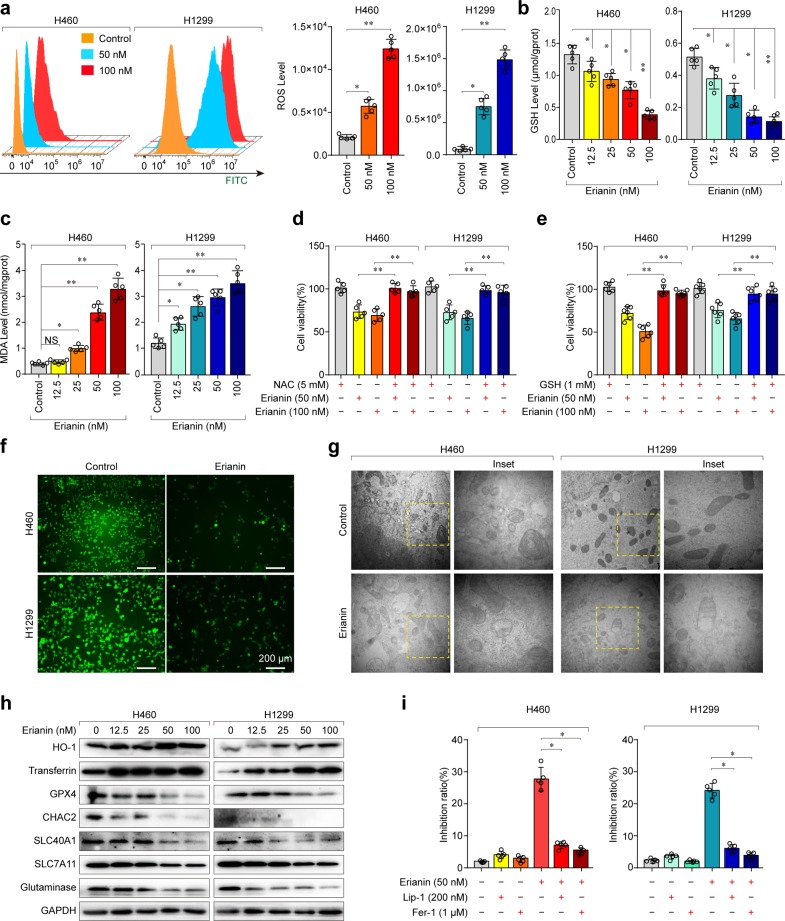
Ferroptosis contributes to erianin-induced cell death in lung cancer cells. **a** The cellular ROS level was analyzed by a flow cytometer, *p < 0.05, **p < 0.01. **b** Intracellular GSH levels in H460 and H1299 cells treated with erianin, *p < 0.05, **p < 0.01. **c** Intracellular MDA levels in H460 and H1299 cells treated with erianin, *p < 0.05, **p < 0.01. **d** H460 and H1299 cells were treated with erianin with or without the ROS scavenger NAC for 24 h, and cell viability was assayed, **p < 0.01. **e** H460 and H1299 cells were treated with erianin with or without the ROS scavenger GSH for 24 h, and cell viability was assayed, **p < 0.01. **f** Intracellular chelatable iron in H460 and H1299 cells treated with erianin was determined using the fluorescent indicator Phen Green SK (green). **g** Transmission electron microscopy (TEM) was used to observe ferroptosis in H460 and H1299 cells (original magnification: ×100). **h** The expression of several key ferroptosis regulators was examined by western blotting. **i** H460 and H1299 cells were treated with erianin with or without ferroptosis inhibitors for 24 h, and the inhibition of growth was assayed, *p < 0.05

It is well known that lipid-reactive oxygen species (ROS) accumulation, lipid peroxidation, and glutathione (GSH) depletion are critical events in ferroptosis.^12^ Therefore, we detected the levels of intracellular ROS, GSH, and the oxidative stress marker malondialdehyde (MDA) in H460 and H1299 cells treated with erianin. As expected, ROS accumulation (Fig. 4a), GSH depletion (Fig. 4b), and lipid peroxidation (Fig. 4c) were significantly increased following treatment with erianin. Furthermore, erianin-induced cell death in lung cancer cells could be rescued by cotreatment with the ROS inhibitors N-acetyl-l-cysteine (NAC) and GSH (Fig. 4d, e).

Iron is an essential reactive element for a variety of biological processes, including ferroptosis. Therefore, we detected intracellular chelatable iron by using the fluorescent indicator Phen Green SK, the fluorescence of which is quenched by iron.^13^ As shown in Fig. 4f, erianin treatment triggered a decrease in the proportion of Phen Green SK-positive cells, indicating that ferroptosis was triggered. For further confirmation, transmission electron microscopy (TEM) was performed. As a result, mitochondrial matrix condensation and the formation of enlarged cristae were found in lung cancer cells treated with erianin (Fig. 4g). Moreover, the expression of HO-1 and transferrin significantly increased after erianin treatment. In contrast, the expression of negative regulatory proteins for ferroptosis (GPX4, CHAC2, SLC40A1, SLC7A11, and glutaminase) significantly decreased after erianin treatment (Fig. 4h). Moreover, erianin-induced cell death in lung cancer cells was almost blocked by cotreatment with the ferroptosis inhibitor liproxstatin-1 (Lip-1) or ferrostatin-1 (Fer-1) (Fig. 4i). Taken together, these findings strongly suggested that erianin triggered ferroptotic cell death in lung cancer cells.

### Calcium/calmodulin signaling contributes to erianin-induced ferroptosis in lung cancer cells

**Fig. 5:**
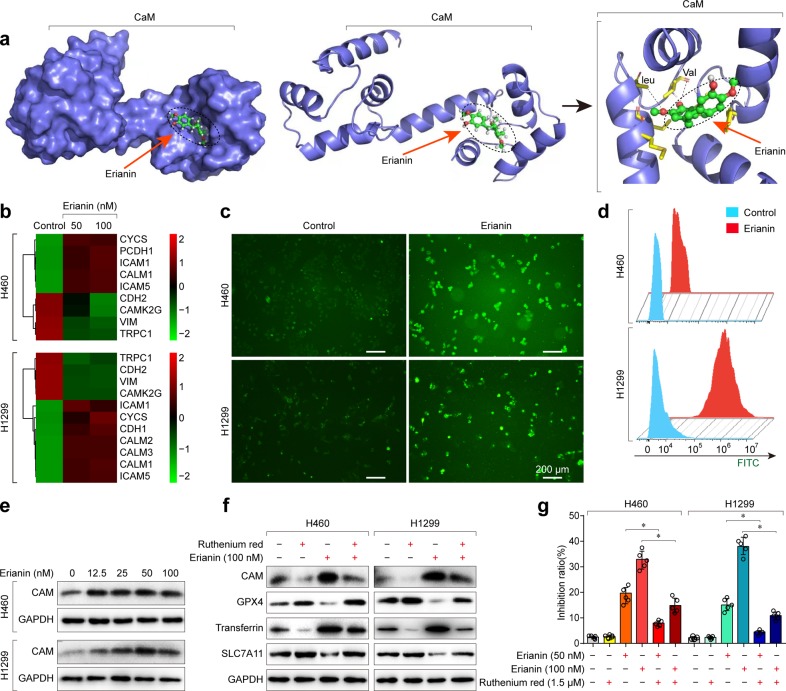
Calcium/calmodulin signaling contributes to erianin-induced ferroptosis in lung cancer cells. **a** The most likely predicted targets of erianin. **b** Heatmap showing the mRNA analysis of Ca^2+^/CaM signaling. **c** The intracellular Ca^2+^ concentration in H460 and H1299 cells treated with erianin was detected according to the fluorescence intensity of the calcium indicator Fluo-3/AM with fluorescence microscopy. **d** The intracellular Ca^2+^ concentration in H460 and H1299 cells treated with erianin was detected by measuring the fluorescence intensity of the calcium indicator Fluo-3/AM with a flow cytometer. **e** The expression of CaM was examined by western blotting after treatment with erianin for 24 h. **f** The effect of blocking Ca^2+^/CaM signaling with ruthenium red on erianin-induced ferroptosis in lung cancer cells after treatment for 24 h. **g** The effect of blocking Ca^2+^/CaM signaling with ruthenium red on erianin-induced inhibition of growth of lung cancer, *p < 0.05

To predict the most likely targets of erianin, the online tool SwissTargetPrediction was utilized for target prediction. According to SwissTargetPrediction, erianin targeted calmodulin (CaM/CALM) (Fig. 5a), which is a major intracellular Ca^2+^-binding protein that controls cell survival and death from fertilization until death. Furthermore, transcriptome analysis also suggested that CaM was activated in lung cancer cells following treatment with erianin (Fig. 5b). To investigate whether Ca^2+^/CaM signaling is a key target for erianin treatment, the intracellular Ca^2+^ concentration was first assessed by detecting the fluorescence intensity of the calcium indicator Fluo-3/AM with a flow cytometer and fluorescence microscopy. As shown in Fig. 5c, d, the Ca^2+^ concentration was significantly elevated in the erianin group compared with that in the control group. The increase in intracellular Ca^2+^ can promote the binding of Ca^2+^ to its regulatory protein. The CaM protein is a main downstream molecule of the calcium signaling pathway. Therefore, we next detected the expression of CaM by western blotting and found that CaM was activated in lung cancer cells in a dose-dependent manner (Fig. 5e). These results indicated that the Ca^2+^/CaM signaling pathway was activated in lung cancer cells after treatment with erianin.

It is well known that CAM can regulate the L-type voltage-dependent Ca^2+^ channel (LVDCC), which is a key transporter involved not only in Ca^2+^ transport but also in iron uptake.^14,15^ Increased Ca^2+^ uptake resulted in ROS production and elevated Fe^2+^ levels.^14,16^ Both ROS accumulation and elevated Fe^2+^ levels caused ferroptosis induction. Therefore, we speculated that erianin could activate CAM and subsequently cause increased Ca^2+^ and Fe^2+^ levels, resulting in ferroptosis induction.

**Fig. 6:**
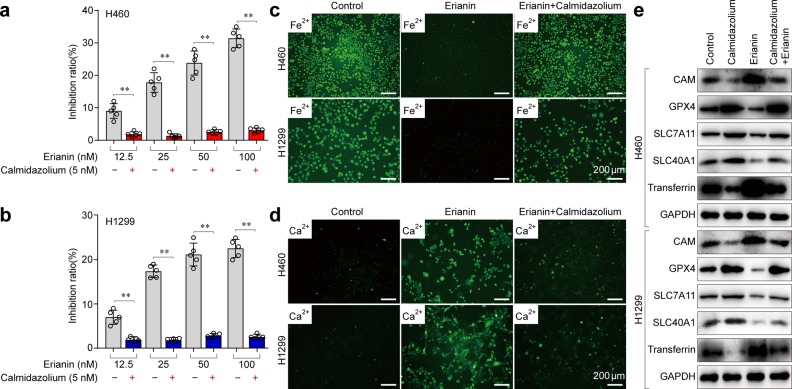
CaM inhibition by calmidazolium rescued erianin-induced ferroptosis. **a** The effect of blocking Ca^2+^/CaM signaling with calmidazolium on erianin-induced inhibition of growth in H460 cells, **p < 0.01. **b** The effect of blocking Ca^2+^/CaM signaling with calmidazolium on erianin-induced inhibition of growth in H1299 cells, **p < 0.01. **c** Intracellular chelatable iron in H460 and H1299 cells after treatment with a combination of calmidazolium and erianin was determined using the fluorescent indicator Phen Green SK (green). **d** The intracellular Ca^2+^ concentration in H460 and H1299 cells after treatment with a combination of calmidazolium and erianin was detected by measuring the fluorescence intensity of the calcium indicator Fluo-3/AM with fluorescence microscopy. **e** Western blotting showed the effect of blocking Ca^2+^/CaM signaling with calmidazolium treatment for 24 h on erianin-induced ferroptosis in lung cancer

To investigate whether Ca^2+^/CaM signaling is a key determinant of erianin-induced ferroptosis, we first treated the lung cancer cells with ruthenium red, an inhibitor of mitochondrial Ca^2+^ uptake and ROS formation. Western blotting showed that blockade of Ca^2+^/CaM signaling significantly decreased the expression of transferrin but increased the abundance of GPX4 and SLC7A11 (Fig. 5f), indicating that Ca^2+^/CaM inhibition resulted in reduced ferroptosis. Furthermore, the CCK-8 assay showed that the inhibition of the growth of H460 cells treated with the combination of ruthenium red and erianin was significantly lower than that of the controls (Fig. 5g). Similar results were obtained in H1299 cells, suggesting that Ca^2+^/CaM signaling induces death in treated cells. Next, we treated these lung cancer cells with the CaM antagonist calmidazolium. Similar to ruthenium red treatment, calmidazolium treatment decreased the erianin-induced inhibition of cell growth (Fig. 6a, b). Meanwhile, decreased Fe^2+^ and Ca^2+^ levels were observed in the lung cancer cells after treatment with the combination of calmidazolium and erianin (Fig. 6c, d). Furthermore, western blotting showed that CaM inhibition by calmidazolium rescued erianin-induced ferroptosis (Fig. 6e).

Taken together, our results demonstrate that Ca^2+^/CaM signaling is a critical regulator of erianin-induced ferroptosis in lung cancer cells.

### Erianin results in ferroptosis induction and exerts antitumor efficacy in vivo

**Fig. 7:**
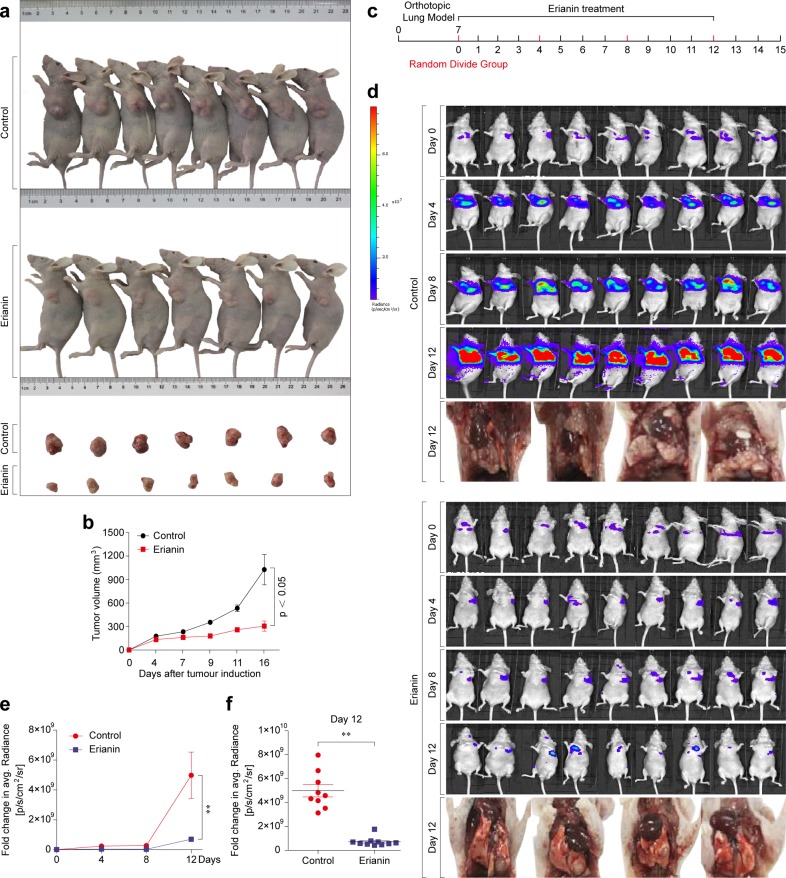
Erianin exerts its antitumor effects in vivo. **a** Representative image of H460 xenograft tumors after erianin treatment. **b** Tumor volume in each group. Data are expressed as the means ± standard deviations (SDs). **c** Scheme of tumor inoculation and systemic injection. **d** Bioluminescent imaging of disseminated H460-luc orthotopic xenograft tumors at different time points posttreatment, and representative images of H460-luc orthotopic xenograft lung tumors after erianin treatment. **e** Fold change in the average radiance per mouse after normalization to the day 0 tumor burden as determined by bioluminescent imaging, **p < 0.01. **f** Fold change in the average radiance per mouse at the experimental endpoint (day 12) for each treatment group (mean ± SEM), **p < 0.01

To estimate the therapeutic potential of erianin in vivo, we combined two types of experimental procedures. First, H460 cells were subcutaneously injected into BALB/c nude mice. When the xenografts reached 100 mm^3^ in size, the mice were randomly divided into two experimental groups: the control group (DMSO) and the erianin group (100 mg/kg). The control groups showed rapid tumor growth, while erianin treatment notably suppressed tumor growth (Fig. 7a, b).

Second, we inoculated the tumor cells directly in the right lung parenchyma to bypass tumor cell homing and to directly assess tumor growth in the lung. Tumors were detected by an IVIS Lumina LT imaging system. In the control groups, the formation of primary tumors at the injection site along with lymph node metastases were significantly observed. Based on the fold change in the bioluminescence of H460-luc cells in mice, erianin significantly inhibited the progression of the lung tumor mass when compared with controls (Fig. 7c–f).

**Fig. 8:**
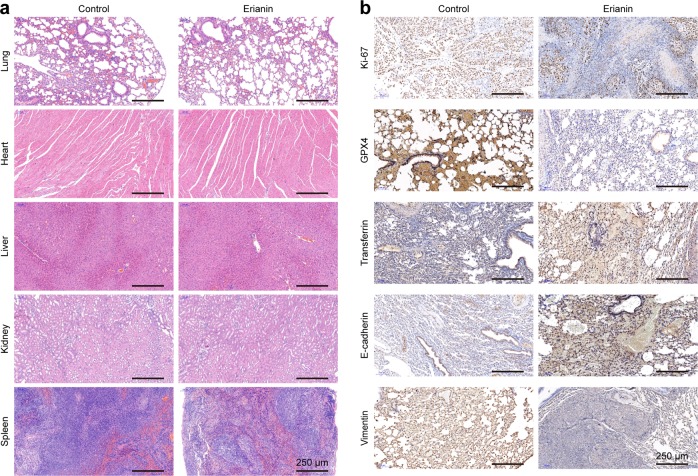
H&E and immunohistochemical staining of xenograft tumor sections. **a** H&E staining of tissue sections of major organs was analyzed 3 days after the last injection of erianin. **b** Immunohistochemical staining of several ferroptotic and metastatic proteins

To assess the in vivo side effects of erianin, various organs were harvested. Organs were sectioned and stained with H&E. No histological differences in the lung, heart, liver, kidney, or spleen were found in the erianin treatment groups, indicating that there was no notable toxicity (Fig. 8a).

We also investigated the expression of Ki-67, GPX4, transferrin, E-cadherin, and vimentin by immunohistochemical staining. As shown in Fig. 8b, the expression of Ki-67 was significantly decreased after erianin treatment, suggesting that erianin could inhibit the growth of lung cancer. Meanwhile, low GPX4 expression and high transferrin expression indicated the occurrence of erianin-induced ferroptosis. Finally, erianin promoted the expression of E-cadherin and suppressed the expression of vimentin. Taken together, our results suggest that erianin inhibited tumor growth in vivo by inducing ferroptosis and suppressed cancer metastasis by inhibiting epithelial–mesenchymal transition.

## Discussion

Previous studies have shown that erianin, a natural product derived from *D. chrysotoxum*, has anticancer activity. However, the role of erianin in lung cancer remains unclear. In this research, we observed the effect of erianin on lung cancer cells both in vitro and in vivo. The results showed that erianin could inhibit cell proliferation, promote G2/M-phase arrest, trigger ferroptosis, and suppress migration in lung cancer cells.

The aim of traditional cancer therapy is to induce cell apoptosis, but many cancer cells are chemoresistant or defective in apoptosis induction. Therefore, developing new agents that enhance different forms of non-apoptotic cell death will hopefully provide a promising therapeutic strategy for cancer patients. Ferroptosis is a recently recognized form of cell death and plays a crucial role in cancer treatment.^17,18^ GPX4 inactivity and subsequent ROS accumulation are central regulators of ferroptosis.^19,20^ Ferroptosis also contributes to the antitumor function of several tumor suppressors, such as p53 and BRCA1-associated protein 1 (BAP1). The p53-mediated transcriptional repression of SLC7A11 contributed to ROS-induced ferroptosis.^21^ BAP1 represses SLC7A11 expression, leading to ferroptosis induction.^22^ Currently, ferroptosis-inducing drugs are attracting more attention for cancer treatment and will hopefully provide a potential strategy for cancer therapy.

**Fig. 9:**
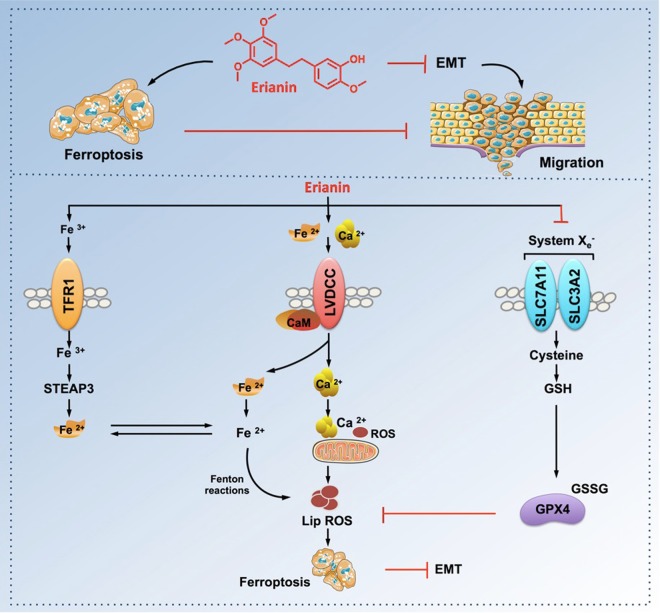
Scheme showing the central role of erianin in ferroptosis induction and inhibition of cell migration

In this research, we reported for the first time that erianin exerted its anticancer activity by inducing ferroptosis, causing G2/M-phase arrest, and inhibiting cell proliferation and metastasis in lung cancer cells. Next, we found that treatment with Z-VAD-FMK, CQ, or necrostatin-1 did not protect against erianin-induced cell death in lung cancer cells, indicating that other forms of cell death may have occurred. Transcriptome analysis indicated that ferroptosis might be a key determinant of erianin-induced cell death. Therefore, we detected whether ferroptosis was present in lung cancer cells during erianin treatment. As expected, ferroptotic events, including ROS accumulation, GSH depletion, and lipid peroxidation, were significantly triggered following treatment with erianin. Moreover, pretreatment with the ferroptosis inhibitor Fer-1, Lip-1, or DFO reduced erianin-induced cell death and suppressed cell migration (Fig. 9). Thus, ferroptosis may contribute to erianin-induced growth inhibition in lung cancer cells.

To determine how erianin induces ferroptosis, the online tool SwissTargetPrediction was first utilized for target prediction, and it was found that erianin might target calmodulin (CaM), a major intracellular Ca^2+^-regulating protein. Transcriptome analysis also suggested that CaM was activated in lung cancer cells following treatment with erianin. Therefore, the Ca^2+^/CaM signaling pathway might be activated in lung cancer cells after treatment with erianin. CAM can regulate the L-type voltage-dependent Ca^2+^ channel (LVDCC), which is key not only for Ca^2+^ transport but also for iron uptake.^14,15^ Increased Ca^2+^ uptake resulted in increased ROS production and elevated Fe^2+^ levels.^14,16^ Both ROS accumulation and elevated Fe^2+^ levels caused ferroptosis induction. Therefore, we speculated that erianin could cause increased Ca^2+^ and Fe^2+^ levels by activating Ca^2+^/CaM signaling. As expected, we demonstrated that erianin triggered ferroptosis in lung cancer cells by activating Ca^2+^/CaM signaling, and the blockage of this signaling significantly decreased cell death caused by erianin treatment by suppressing ferroptosis (Fig. 9).

In summary, our research demonstrated that erianin might be a new ferroptosis inducer and has the potential to induce cell death and inhibit metastasis in lung cancer cells. Our data will hopefully support the use of erianin as a potential compound for lung cancer treatment. To maximize the potential of erianin for application in the clinic, large-scale, multicenter collaborative clinical trials will be urgently needed in the future.

## Methods

### Cell lines and reagents

The human lung cancer H460 and H1299 cell lines were purchased from ATCC (LGC Standards SLU, Barcelona, Spain). The cell lines were maintained in Roswell Park Memorial Institute (RPMI)-1640 medium with 10% fetal bovine serum (FBS) at 37 °C in a humidified atmosphere of 95% air and 5% CO_2_. Purified erianin (>98%) (#95041) was purchased from Shanghai Yuanye Biological Co., Ltd. The 25 mM stock solution was made in DMSO (#D8371) purchased from Solarbio Science & Technology Co., Ltd.

Deferoxamine (#HY-B0988), ferrostain-1 (#HY-100579), necrostain-1 (#HY-15760), N-acetyl-l-cysteine (NAC) (#616-91-1), and glutathione (GSH) (#HY-D0187/CS-7948) were purchased from MCE. Z-VAD-FMK (#V116), chloroquine (#C6628), and calmidazolium (APN16136-1-6) were obtained from Sigma Aldrich. Liproxstatin-1 (#S7699) was purchased from Selleck Chemical. Proteins were reacted with one of the following: GPX4 (#GR251529-34), HO-1 (#GR3187585-3), glutaminase (#GR3299063-1), SLC40A1 (#GR215168-39), transferrin (#GR3207592-9), SLC7A11 (#GR3235736-6), and anti-calmodulin 1/2/3 (ab45689); all of these were purchased from Abcam. N-Cadherin (#13116S), E-cadherin (#14472S), MMP-9 (#13667S), snail (#3879T), slug (#9585T), and GAPDH (#2118S) were purchased from Cell Signaling Technology (CST). CHAC2 (YB-13883R) was obtained from Ybscience. Vimentin (#SA10106DB) was obtained from ABGENT. Ki-67 (#66434-1-Ig) was purchased from Proteintech.

### Measurement of the cell inhibition rate and apoptosis

The cell inhibition rate was evaluated using a cell counting kit-8 (CCK-8) assay. A Phartmingen Annexin V–FITC Apoptosis Detection Kit I (BD, USA) was utilized to detect cell death by flow cytometry.

### Transwell and wound-healing assays

For the Transwell migration assay, 1 × 10^5^/ml cells were suspended in the upper chamber of a 24-well Transwell plate. After 24 h of coculture, the cells were fixed, stained, and counted under a Nikon light microscope (Nikon Corporation).

For the wound-healing assay, a monolayer of cells at 95% confluence was scratched with a sterile plastic tip and then cultured in serum-free medium.

### Colony-formation assays

For the colony-formation assays in monolayer cultures, cells (1 × 10^3^/well) were plated in a 10 cm dish for 2 weeks. After fixation and staining, the colonies were imaged and counted.

### Measurement of ROS

The peroxide-sensitive fluorescent probe DCFH–DA (#S0033) was used to detect intracellular ROS according to the manufacturer’s instructions.

### Malondialdehyde (MDA) assay

MDA is a major indicator of lipid peroxidation, which was detected and normalized on the basis of the protein concentration according to the manufacturer’s instructions.

### GSH assay

The total quantities of glutathione were measured using a GSH Assay Kit (#A006-2-1) purchased from Nanjing Jiancheng and normalized on the basis of the cell number according to the manufacturer’s instructions.

### Immunohistochemistry

Tumor tissues were fixed, paraffin-embedded, and sectioned (4 μm). The deparaffinized and rehydrated sections were subjected to antigen retrieval using sodium citrate buffer. After incubation with 10% normal goat serum for 1 h, the sections were incubated with primary antibody overnight at 4 °C. The subsequent procedures were performed according to the manufacturer’s instructions.

### RNA library construction and sequencing

The total RNA was extracted by TRIzol reagent (Invitrogen, CA, USA). Then, we performed paired-end sequencing on an Illumina HiSeq 4000 at LC-BIO Technologies (Hangzhou) Co., LTD. by following the vendor’s recommended protocol.

### In vivo tumor model

All of the in vivo experimental protocols were approved by the animal care committee of Hangzhou Normal University. H460-luc cells (2 × 10^6^ cells in 0.1 ml phosphate-buffered saline) were subcutaneously injected into the right dorsal flank of 6-week-old female BALB/c nude mice; for the orthotopic xenograft lung tumor mouse model, a volume of 100 μl of cell suspension was injected into the right side of the lung. Tumor volume was assessed every 2 days and was calculated by the following formula: (short diameter)^2^ × (long diameter)/2. The presence of the orthotopic xenograft lung tumor was confirmed by the IVIS Lumina LT imaging system. When tumor formation was confirmed, the mice were randomly divided into two groups: the control group and the erianin treatment group. After 15 days of drug administration (intraperitoneal injection, once daily), the mice were killed, and samples were obtained for the immunohistochemical experiments.

### Statistical analyses

The significance of differences between groups was determined using a *t* test. Statistical analysis was performed using GraphPad Prism 7.0c Software. A *p*-value < 0.05 was considered statistically significant.

## Supplementary information

Supplementary Materials
