# Supplementary material for: Erianin, a novel dibenzyl compound in Dendrobium extract, inhibits lung cancer cell growth and migration via calcium/calmodulin-dependent ferroptosis
Source: Signal Transduct Target Ther. 2020 May 8;5:51. doi: 10.1038/s41392-020-0149-3 (PMC7205607; doi:10.1038/s41392-020-0149-3)
Supplement: Supplementary file 1 — Supplementary Materials [file 41392_2020_149_MOESM1_ESM.docx]

Supplementary Materials for

**Erianin, a novel dibenzyl compound in Dendrobium extract, inhibits lung cancer cell growth and migration via calcium/calmodulin-dependent ferroptosis**

Peng Chen^1, 2, 4, 5, †^, Qibiao Wu^5, †^, Yu Xiang^1, 3, 4^, Mingming Zhang^1, 4^, Jiao Feng^1, 4^, Shuiping Liu^1, 4^, Ting Duan^1, 4^, Lijuan Zhai^1, 3, 4^, Bingtao Zhai^1, 4^, Wengang Wang^1, 4^, Ruonan Zhang^1, 4, 5^, Liuxi Chen^1^, Bi Chen^1, 4, 5^, Xuemeng Han^1, 4^, Xiaying Chen^1, 4^, Yicong Li^6^, Ying Liu^7^, Xingxing Huang^1^, Ting Pan^1, 4^, Lili Yan^1, 4^, Ting Jin^1, 4^, Wenzheng Zhang^1^, Hong Luo^1^, Xiaohui Chen^1^, Yongqiang Li^1^, Qiujie Li^1, 4^, Guohua Li^1, 4^, Qin Zhang^1, 4^, Lvjia Zhuo^1, 4^, Zuyi Yang^9^, Huifen Tang^9^, Tian Xie^1,^ ^4, *^, Xiaoping Ouyang^8, *^, Xinbing Sui^1,^ ^4, 10, *^

†These authors contributed equally to this work

Correspondence to: Tian Xie, Email: xbs@hznu.edu.cn or Xiaoping Ouyang, Email: droyxp@aliyun.com or Xinbing Sui, Email: hzzju@zju.edu.cn

Figure. S1


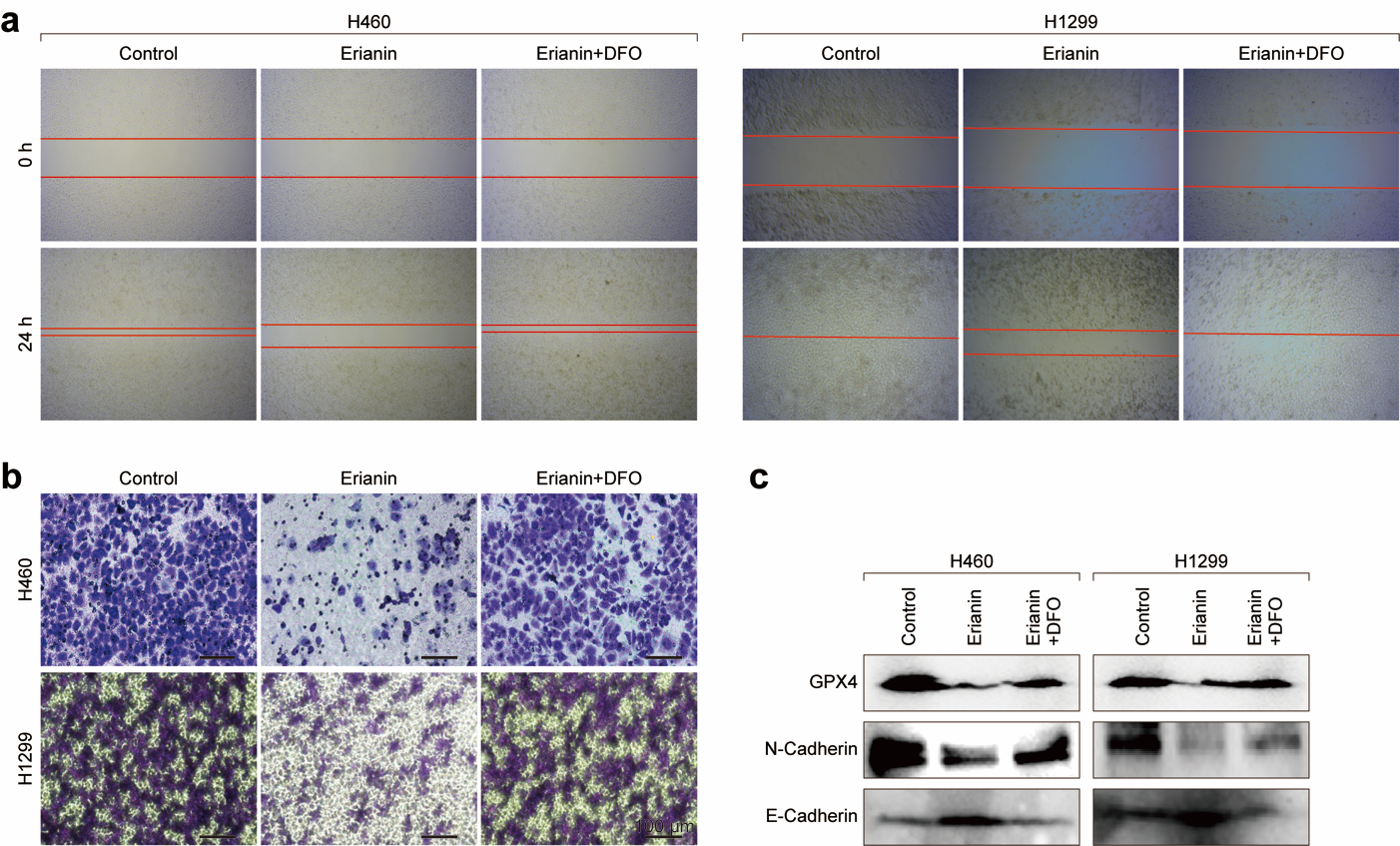


**Figure S1. Inhibition of Erianin-induced ferroptosis promoted the migration of lung cancer cells. a**, Representative results of wound healing after the treatment with the combination of ferroptosis inhibitor DFO and erianin. **b**, Transwell migration assay by the 24-transwell system and quantitative analysis. The pictures were taken 24 h after seeding (original magnification: × 100). **c**, The expression of EMT markers E-Cadherin and N-Cadherin were examined by western blotting.

Figure. S2

**
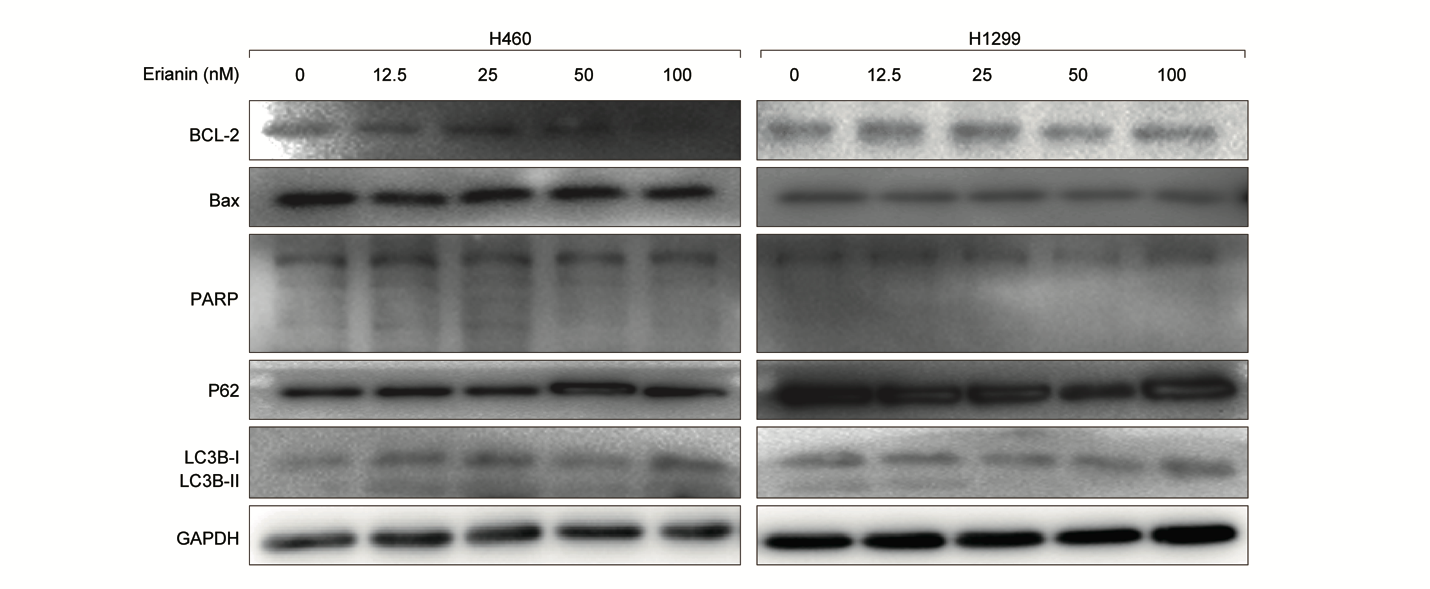
**

**Figure S2. The effect of erianin on apoptosis and autophagy determined by western blotting. GAPDH was used as internal control.**
